# Transformation Kinetics of LiBH_4_–MgH_2_ for Hydrogen Storage

**DOI:** 10.3390/molecules27207005

**Published:** 2022-10-18

**Authors:** Ou Jin, Yuanyuan Shang, Xiaohui Huang, Dorothée Vinga Szabó, Thi Thu Le, Stefan Wagner, Thomas Klassen, Christian Kübel, Claudio Pistidda, Astrid Pundt

**Affiliations:** 1 Institute for Applied Materials, Karlsruhe Institute of Technology, 76131 Karlsruhe, Germany; 2 Institute of Nanotechnology, Karlsruhe Institute of Technology, 76344 Eggenstein-Leopoldshafen, Germany; 3 Institute of Hydrogen Technology, Helmholtz-Zentrum Hereon GmbH, 21502 Geesthacht, Germany; 4 Karlsruhe Nano Micro Facility, Karlsruhe Institute of Technology, 76344 Eggenstein-Leopoldshafen, Germany; 5 Joint Research Laboratory Nanomaterials, Technical University of Darmstadt, 64206 Darmstadt, Germany

**Keywords:** hydrogen storage, transmission electron microscopy, crystallography, reactive hydride composite, additive, phase transformation

## Abstract

The reactive hydride composite (RHC) LiBH_4_–MgH_2_ is regarded as one of the most promising materials for hydrogen storage. Its extensive application is so far limited by its poor dehydrogenation kinetics, due to the hampered nucleation and growth process of MgB_2_. Nevertheless, the poor kinetics can be improved by additives. This work studied the growth process of MgB_2_ with varying contents of 3TiCl_3_·AlCl_3_ as an additive, and combined kinetic measurements, X-ray diffraction (XRD), and advanced transmission electron microscopy (TEM) to develop a structural understanding. It was found that the formation of MgB_2_ preferentially occurs on TiB_2_ nanoparticles. The major reason for this is that the elastic strain energy density can be reduced to ~4.7 × 10^7^ J/m^3^ by creating an interface between MgB_2_ and TiB_2_, as opposed to ~2.9 × 10^8^ J/m^3^ at the original interface between MgB_2_ and Mg. The kinetics of the MgB_2_ growth was modeled by the Johnson–Mehl–Avrami–Kolmogorov (JMAK) equation, describing the kinetics better than other kinetic models. It is suggested that the MgB_2_ growth rate-controlling step is changed from interface- to diffusion-controlled when the nucleation center changes from Mg to TiB_2_. This transition is also reflected in the change of the MgB_2_ morphology from bar- to platelet-like. Based on our observations, we suggest that an additive content between 2.5 and 5 mol% 3TiCl_3_·AlCl_3_ results in the best enhancement of the dehydrogenation kinetics.

## 1. Introduction

In the context of a low-carbon economy and the present global energy crisis, there is a rapidly growing demand for sustainable energy based on renewable energy sources, e.g., solar, wind power, and hydroelectric power [1]. Countless studies have been devoted to storing power from these intermittent sources. In addition to electrical energy storage, another important option is to store energy in the form of hydrogen in various media [2,3,4]. Hydrogen as a zero-carbon fuel can deliver an excellent energy density of approximately 120 MJ/kg. Currently, the most commercially utilized way to store hydrogen is by compressing hydrogen under ultrahigh pressure up to several hundred bars or by liquefying hydrogen below its boiling point down to −253 °C under atmospheric pressure and keeping it in specifically designed vessels [5,6]. However, harsh conditions are still necessary for both approaches, which will inevitably reduce the public acceptance of the extensive application of hydrogen. Therefore, storing hydrogen in solid-state materials is commonly regarded as the promising form of hydrogen storage [7].

LiBH_4_–MgH_2_ is one of the promising reactive hydride composites for hydrogen storage [8,9,10]. By combining the complex metal hydride LiBH_4_ and the metal hydride MgH_2_, the standard dehydrogenation enthalpy of the mixture is remarkably lowered by ~25 kJ·mol^−1^ H_2_ [11,12,13]. This should lead to a dehydrogenation temperature of about 170 °C, with an excellent hydrogen storage capacity of about 11.5 wt% still reserved. According to preceding studies, the dehydrogenation of LiBH_4_–MgH_2_ occurs in two steps [14]. First, MgH_2_ decomposes into H_2_ and Mg. Then, the generated Mg reacts with LiBH_4_ to produce MgB_2_ and LiH, releasing the remaining H_2_ from LiBH_4_. However, after the first step of the reaction, it can take more than 10 h until the second step begins, which is not acceptable for commercial utilization [15]. The long waiting time between the two reaction steps has been attributed to the sluggish nucleation process of MgB_2_ [16]. It was later reported that this obstacle can be overcome by some transition metal additives, which notably accelerate the kinetics [14,17]. Bösenberg et al. suggested that the involvement of these additives may create more coherent nucleation sites promoting the nucleation of MgB_2_ by lowering the interfacial strain energy [18]. This hypothesis was later experimentally validated by Jin et al., who additionally distinguished between two different MgB_2_ morphologies by transmission electron microscopy (TEM) [19]. They considered the generation of MgB_2_ platelets assisted by additives rather than the generation of MgB_2_ bars based on Mg as an indication of a small interatomic misfit between the two phases, which is the origin of the improved kinetic performance. However, the additive effect on the subsequent growth process for MgB_2_ has not been fully clarified yet. This knowledge would be another essential cornerstone of building up a comprehensive understanding of the transformation kinetics of LiBH_4_–MgH_2_.

In this work, varying amounts of 3TiCl_3_·AlCl_3_ additives were added to LiBH_4_–MgH_2_ to study their impact on the MgB_2_ formation after dehydrogenation using advanced TEM techniques. The applied TEM techniques include electron energy loss spectroscopy (EELS), 4D scanning transmission electron microscopy (4DSTEM) and electron tomography etc. The TEM results have been combined with the results from kinetic measurements and X-ray diffraction (XRD) for further analysis.

## 2. Results and Discussion

### 2.1. Material Characterization by XRD and Kinetic Performance

**Figure 1:**
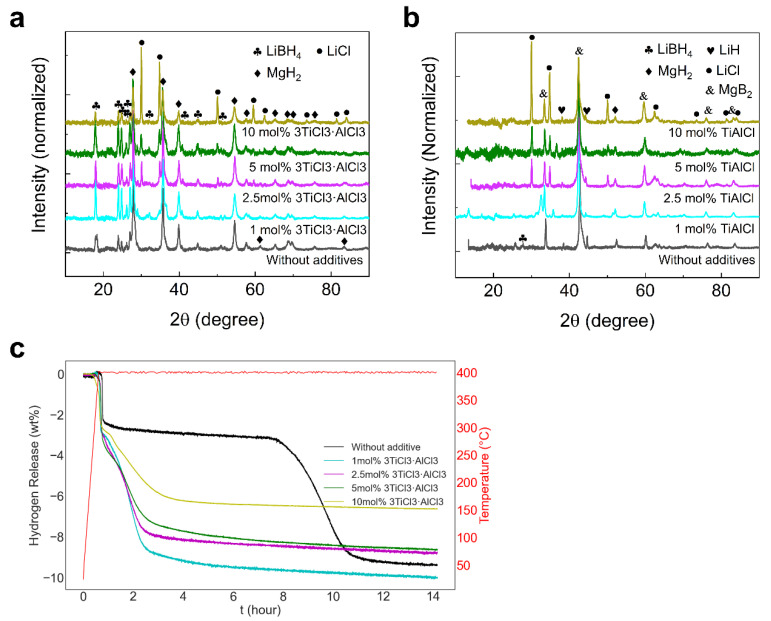
(**a**) XRD patterns of as-milled 2LiBH_4_–MgH_2_ with x mol% 3TiCl_3_·AlCl_3_ (x = 0, 1, 2.5, 5 and 10); (**b**) XRD patterns of desorbed 2LiBH_4_–MgH_2_ with x mol% 3TiCl_3_·AlCl_3_ (x = 0, 1, 2.5, 5 and 10); (**c**) Measurements of dehydrogenation kinetics for 2LiBH_4_–MgH_2_ with x mol% 3TiCl_3_·AlCl_3_ (x = 0, 1, 2.5, 5 and 10) at 400 °C and under 4 bar H_2_, showing the improved dehydrogenation kinetics by the additives.

Figure 1a shows the XRD diffractograms of the samples with varying contents of 3TiCl_3_·AlCl_3_ additive in the as-milled state. Different peaks were detected, marking the reactants LiBH_4_ and MgH_2_. In addition to this, LiCl can also be identified in the diffractogram when the additive content exceeds 2.5 mol%. This by-product is generated from the reaction between LiBH_4_ and 3TiCl_3_·AlCl_3_ during the ball milling. With 10 mol% additives, the LiCl peaks are significantly enhanced, whereas the peaks for LiBH_4_ are greatly weakened. This happens as LiBH_4_ is consumed by the additive [20]. For the desorbed samples, the diffraction peaks of the LiBH_4_ and MgH_2_ were not observed, see Figure 1b, while the peaks of LiH and MgB_2_ appeared. This indicates that the reaction between LiBH_4_ and MgH_2_ proceeded with the generation of MgB_2_.

Another way to verify that the dehydrogenation process of LiBH_4_–MgH_2_ is completed is to monitor the amount of hydrogen released from the material over time. As shown in Figure 1c, the more additive that was added to the system, the less hydrogen was released, which is consistent with the intensified LiCl peaks shown in Figure 1a,b. This means that less LiBH_4_ is available for the reaction with MgH_2_. One anomaly is that the sample without additives only released less than 10 mol% H_2_, which is below the expectation of a theoretical value of 11.5 mol% H_2_. This can be attributed to the partial oxidation of LiBH_4_ or/and MgH_2_, the low purity of the raw materials from the suppliers, or the inhomogeneous dispersion of reactants over ball milling, and so on. It is also notable that the additive improves the dehydrogenation kinetics very effectively. In contrast to the curve with respect to hydrogen release from the sample without additives, the incubation stage almost entirely disappeared with the addition of only 1 mol% 3TiCl_3_·AlCl_3_. In general, the duration of the dehydrogenation process is shortened from about 12 h without additives to about 4 h with additives. Furthermore, these curves seem to have different sigmoidal patterns, which might relate to the different MgB_2_ growths during the reaction between LiBH_4_ and Mg.

### 2.2. Observation of MgB_2_ Using TEM

**Figure 2:**
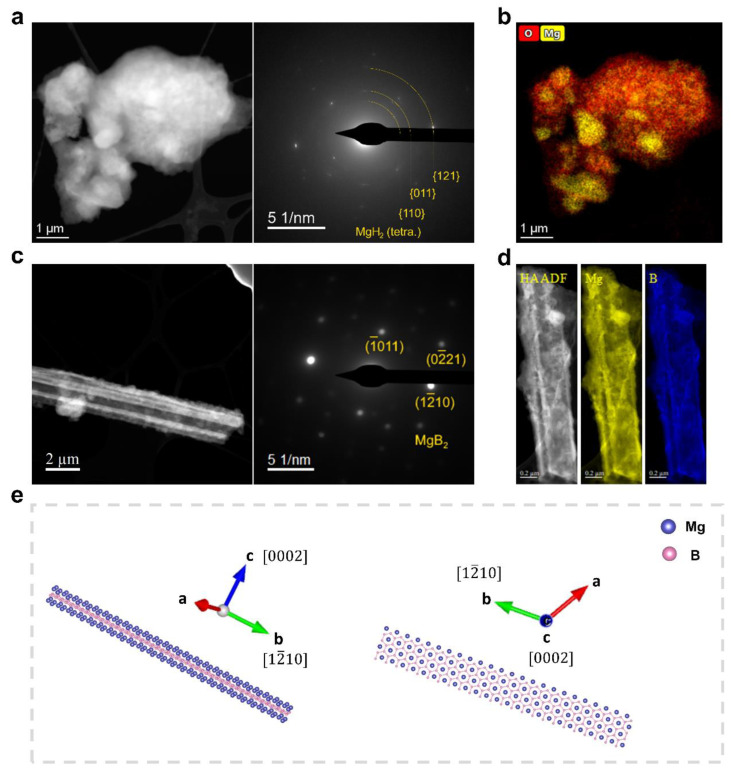
As-milled or desorbed 2LiBH_4_–MgH_2_ without additive: (**a**) STEM-HAADF image and the corresponding DP of as-milled 2LiBH_4_–MgH_2_; (**b**) Summed EDX elemental map that was acquired in the same position as image (**a**), comprising the elemental distribution of Mg (K lines) and O (K lines); (**c**) STEM-HAADF image and the corresponding DP of desorbed 2LiBH_4_–MgH_2_; (**d**) EELS elemental distribution of Mg (K edge) and B (K edge) with respect to the corresponding STEM-HAADF image of desorbed 2LiBH_4_–MgH_2_ shows the bar-like morphology for MgB_2_; (**e**) Schematic illustration of the crystallographic orientations for a MgB_2_ rectangular bar growing in the direction $\left[1\bar{2}10\right]$.

Figure 2a exhibits the morphology of the reactants LiBH_4_–MgH_2_ prior to dehydrogenation. The corresponding diffraction pattern shows the diffraction spots representing MgH_2_. The missing crystallographic information for LiBH_4_ in the diffraction pattern can presumably be attributed either to oxidation or to electron beam damage, as LiBH_4_ is both air- and electron-beam-sensitive [21,22]. This speculation can be to some extent confirmed by Figure 2b, which shows the same region as Figure 2a, and delivers the elemental distribution of magnesium and oxygen, as obtained by local EDX. The distribution of Mg directly represents the distribution of MgH_2_, since Mg only exists as MgH_2_ in this region. What’s interesting is that the MgH_2_ grains are embedded in some of the oxygen-containing material. It is thus reasonable to assume that this material corresponds to oxidized LiBH_4_ present in the surrounding area.

Figure 2c exhibits the STEM-HAADF image of the material after dehydrogenation of LiBH_4_–MgH_2_. The diffraction pattern was taken from the corresponding 4D-STEM data stack. Accordingly, the crystals growing in the same direction are MgB_2_, and their growth direction is $\left[1\bar{2}10\right]$. This is in agreement with previous studies [19]. The uniform distribution of magnesium and boron recorded via STEM-EELS (Figure 2d) further proves the formation of MgB_2_ with a bar-shaped morphology. Given the crystal structure and the rectangular bar-like morphology of MgB_2_, the other two directions that are vertical to $\left[1\bar{2}10\right]$ were determined to be $\left[10\bar{1}0\right]$ and [0002], see Figure 2e. The disappearance of LiH may be due to electron beam damage [23].

**Figure 3:**
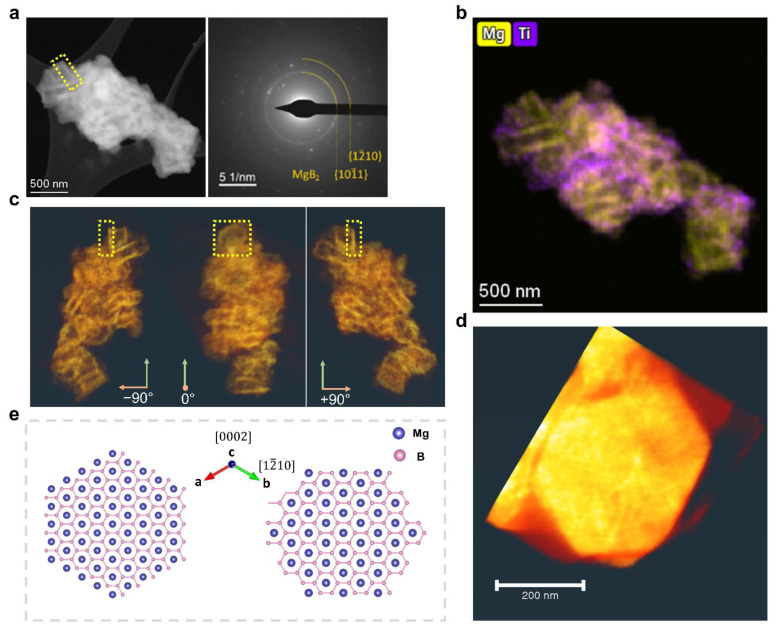
Desorbed 2LiBH_4_–MgH_2_ with 10 mol% 3TiCl_3_·AlCl_3_: (**a**) STEM-HAADF image and the corresponding DP; (**b**) Summed EDX elemental map of Mg (K lines) and Ti (K lines) acquired in the same position as image a; (**c**) Tomography images based on the dataset of EDX map of Mg (K lines) acquired in the same region as image a visualized at the angle −90°, 0° and +90°. The MgB_2_ platelet highlighted in the yellow box is exactly the one highlighted in image (**a**); (**d**) 3D visualization from tomographic reconstruction of the selected MgB_2_ crystal indicated in images (**a**,**c**) shows a hexagonal platelet; (**e**) Schematic illustration of the crystallographic orientations for a MgB_2_ hexagonal platelet in the zone axis [0002].

For a more detailed comparison, we first focused on the extreme case where 10 mol% 3TiCl_3_·AlCl_3_ was added, and then continued with the results on samples with a lower additive content. Figure 3a exhibits the desorbed LiBH_4_–MgH_2_ with 10 mol% 3TiCl_3_·AlCl_3_, with the major crystal also being MgB_2_ according to the diffraction pattern. Figure 3b displays the EDX map of Mg and Ti, showing the distribution of MgB_2_ and Ti-containing materials. Similar to the previous observations without additives, there are parallel MgB_2_ crystals oriented in the same direction, yet with a much smaller size. To determine the MgB_2_ morphology in this case, STEM tomography analyses were carried out on the region shown in Figure 3a. Figure 3c displays the tomography images reconstructed from the EDX map of Mg at different angles. One exemplarily selected piece of MgB_2_ in the yellow box marked in both Figure 3a,c was further studied; see Figure 3d. In contrast to the bar-like MgB_2_ crystals displayed in Figure 2c,d, a regular hexagonal platelet appeared in the case of the 10 mol% additive. As illustrated in Figure 3e, given the hexagonal close-packed (hcp) crystal structure of MgB_2_, it can be immediately determined that this corresponds to the basal plane {0002} [24]. The six-fold symmetrical surface planes can also be indexed. The two possible candidates for these planes are the primary prism plane $\left\{10\bar{1}0\right\}$ and the second prism plane $\left\{1\bar{2}10\right\}$, as shown in Figure 3e. According to recent studies, the bar-like MgB_2_ crystals nucleate on Mg grains, whereas the nucleation of platelet-like MgB_2_ occurs on TiB_2_ nanoparticles [19]. Based on the edge-to-edge matching model [25,26,27], their respective orientation relationships and the corresponding misfits have been previously determined, which are summarized in Table 1.

With this information, we can calculate the elastic strain energy densities $\epsilon_{\mathrm{hkil}}$ for any given lattice plane hkil with the equation $\epsilon_{\mathrm{hkil}}=\frac{1}{2}{\;Y}_{\mathrm{hkil}}\delta_{\mathrm{hkil}}^{2}$, where $Y_{\mathrm{hkil}}$ and $\delta_{\mathrm{hkil}}$ represent the Young’s modulus and the atomic misfit in a certain direction, respectively [28]. The corresponding Young’s modulus $Y_{\mathrm{hkil}}$ for MgB_2_ can be then calculated by substituting the Miller indices of the lattice plane hkil, the elastic constants $C_{11}$ = 365 GPa, $C_{12}$ = 98 GPa, $C_{13}$ = 65 GPa, $C_{33}$ = 203 GPa and $C_{44}$ = 58 GPa, and the lattice constants a = 3.0851 Å and c = 3.5201 Å into Equation (1) [29,30]. The compliances $S_{\mathrm{ij}}$ in the equation can be transferred from the elastic constants $C_{\mathrm{lk}}$ based on the given crystal structure [31].

(1) $$Y_{\mathrm{hkil}}=\frac{{\left[h^{2}+\frac{{\left(h+2k\right)}^{2}}{3}+{\left(\frac{a}{c}l\right)}^{2}\right]}^{2}}{\left[S_{11}{\left(h^{2}+\frac{{\left(h+2k\right)}^{2}}{3}\right)}^{2}+{\;S}_{33}{\left(\frac{a}{c}l\right)}^{4}+\left(S_{44}+2S_{13}\right)\left(h^{2}+\frac{{\left(h+2k\right)}^{2}}{3}\right){\left(\frac{a}{c}l\right)}^{2}\right]}$$

Combining the calculated Young’s modulus $Y_{\mathrm{hkil}}$ for the selected orientations and the related misfit $\delta_{\mathrm{hkil}}$ from Table 1 gives the elastic strain energy densities $\epsilon_{\mathrm{hkil}}$ for MgB_2_; see Table 2.

According to the experimental observations and the determined elastic strain energy presumably induced during the formation of MgB_2_, the large energy density of more than 7 × 10^8^ J/m^3^ along the 〈0002〉 direction explains why both MgB_2_ bars (on Mg) and MgB_2_ platelets (on TiB_2_) appeared to be rather thin in this direction. For Mg, the nucleation and growth of MgB_2_ along the $\langle 10\bar{1}0\rangle$ direction on Mg also led to a significant amount of strain energy density of up to 3·10^8^ J/m^3^. The related high strain energy at the interface may account for the primary growth along the $\langle 1\bar{2}10\rangle$ direction, which is perpendicular to the interface between MgB_2_ and Mg, leading to the morphology of a rectangular bar. In contrast for TiB_2_, the nucleation of MgB_2_ along either $\langle 10\bar{1}0\rangle$ or $\langle 1\bar{2}10\rangle$ on TiB_2_ is equivalent from an energetic point of view, leading to a significantly smaller strain energy density of only 4.7·10^7^ J/m^3^. This value is about an order of magnitude smaller than that of the corresponding growth on Mg, which explains the more isotropic morphology of the hexagonal MgB_2_ platelets. MgB_2_ bars are also distinct from MgB_2_ platelets in terms of their aspect ratio, which is much larger than one, indicating that they grow predominantly in one direction.

Another interesting point is the observation of a parallel alignment of the MgB_2_ crystals observed in both cases (with or without additives). We can understand where the parallel alignment of the MgB_2_ bars has come from, as Mg grains are large enough to provide sufficient surface area for the nucleation of several MgB_2_ bars on the same plane; see Figure 2b. Since the nucleation of MgB_2_ follows a specific crystallographic orientation with respect to Mg, it is natural for the MgB_2_ crystals nucleating on the same Mg plane to grow in the same direction. However, given the distance between two parallel MgB_2_ platelets up to several hundred nanometers (Figure 3a), and the size of TiB_2_ nanoparticles (Figure 3b), it is not likely for two MgB_2_ platelets to nucleate and grow on the same TiB_2_ nanoparticle. From this perspective, one assumption is that some TiB_2_ nanoparticles may be attached to Mg grains in certain orientations with respect to Mg to minimize their interfacial energy during the dehydrogenation process. The nucleation of MgB_2_ is then more likely to first occur on these attached nanoparticles. This is not only because the nucleation of MgB_2_ based on TiB_2_ requires less strain energy per unit volume, but also because the diffusion distance for Mg atoms is much shorter, as these TiB_2_ nanoparticles are directly attached on the surface of Mg grains.

**Figure 4:**
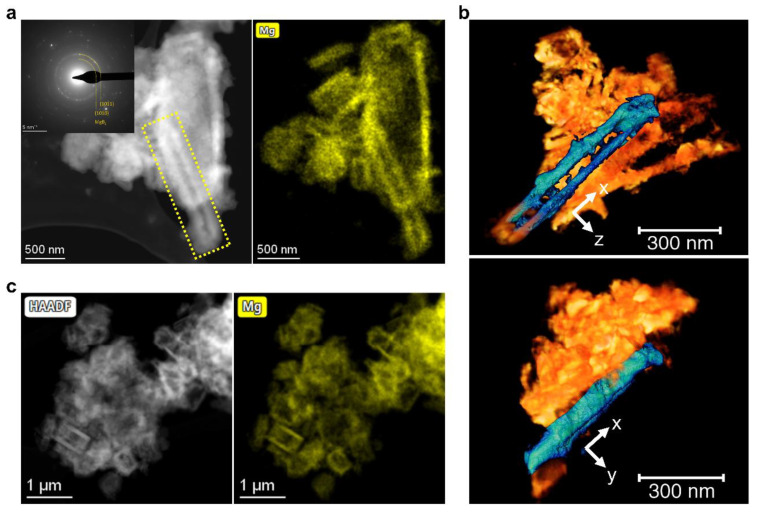
Desorbed 2LiBH_4_–MgH_2_ with 1 mol% 3TiCl_3_•AlCl_3_: (**a**) STEM-HAADF image, and the corresponding DP and EDX elemental map of Mg (K lines), where shows a MgB_2_ morphology similar to that of Figure 2; (**b**) Volume rendering from tomographic reconstruction of the parallel MgB_2_ bars (highlighted in blue) of the following: (**c**) a STEM-HAADF image and the corresponding EDX map of Mg (K lines) showing the similar morphology of MgB_2_ as that of Figure 3.

To understand the dependence of the MgB_2_ morphology on the additive content, samples added with lower contents of 3TiCl_3_·AlCl_3_ were also studied. Figure 4a shows a STEM-HAADF image of the sample with 1 mol% additives after dehydrogenation. Again, the corresponding diffraction pattern confirms the existence of MgB_2_. By comparing this image with the corresponding elemental distribution of Mg, the parallel-oriented crystals of MgB_2_ can be recognized in the agglomerate. As indicated in Figure 4a, two parallel MgB_2_ crystals were selected for electron tomography measurements. It turns out that both pieces of MgB_2_ have a bar-like morphology (Figure 4b). In some other regions, a MgB_2_ morphology similar to Figure 3 can also be observed, which indicates the generation of MgB_2_ platelets (Figure 4c). These observations indicate that the nucleation of MgB_2_ on Mg has occurred. Besides, the nucleation of MgB_2_ on TiB_2_ was not as dominant as in the case of 10 mol% additives, where no more MgB_2_ bars were observed. This also implies that there is a competition between the nucleation on Mg grains and TiB_2_ nanoparticles for Mg and B atoms to generate MgB_2_ bars or MgB_2_ platelets.

**Figure 5:**
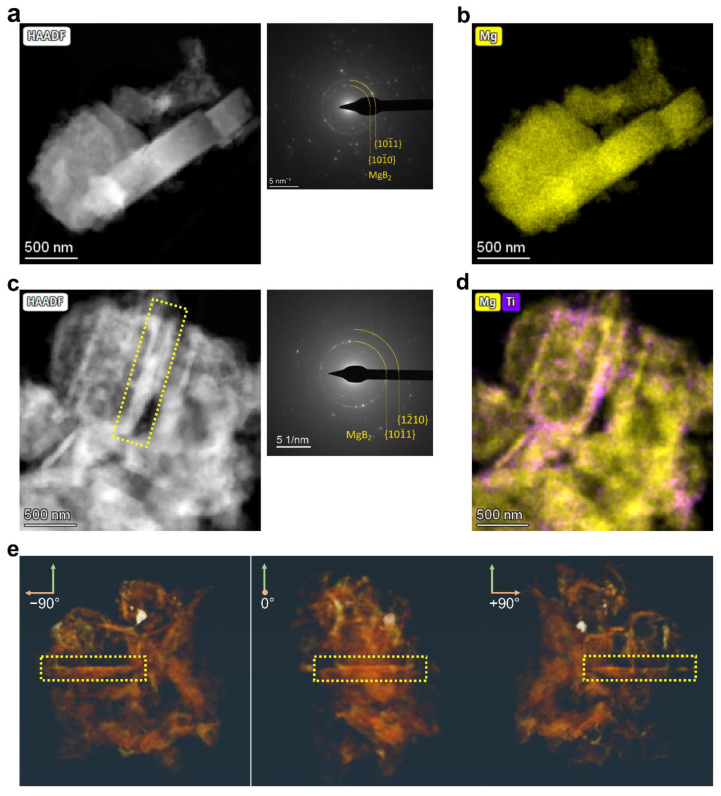
Desorbed 2LiBH_4_–MgH_2_ with 2.5 mol% or 5 mol% 3TiCl_3_·AlCl_3_: (**a**) STEM-HAADF image and the corresponding DP of desorbed 2LiBH_4_–MgH_2_ with 2.5 mol% 3TiCl_3_·AlCl_3_; (**b**) EDX elemental map of Mg (K lines) acquired in the same position as image (a); (**c**) STEM-HAADF image and the corresponding DP of desorbed 2LiBH_4_–MgH_2_ with 5 mol% 3TiCl_3_·AlCl_3_; (**d**) Summed EDX elemental map of Mg (K lines) and Ti (K lines) acquired in the same position as image (**c**); (**e**) STEM tomography images acquired in the same region as image (**c**) visualized at the angle −90°, 0° and +90°. The selected MgB_2_ platelet highlighted in the yellow box is exactly the one highlighted in image (**c**).

When the additive content was raised up to 2.5 mol%, we still observed MgB_2_ bars in some areas (Figure 5a,b). After further increasing the additive content to 5 mol%, the majority of the observed MgB_2_ crystals were platelet-like, as shown in Figure 5c,d. This can be further verified by the tomography analysis on the piece of MgB_2_ highlighted in the yellow box in Figure 5c. These tomography images display a MgB_2_ platelet at different angles. Based on these observations, it seems to imply a turning point in the competition between the nucleation on Mg and TiB_2_.

### 2.3. Kinetic Modeling

According to the TEM results, when varying contents of 3TiCl_3_·AlCl_3_ are added to LiBH_4_–MgH_2_, the dehydrogenation processes of LiBH_4_–MgH_2_ differ. This difference is not only reflected in the change in MgB_2_ morphology, but also in the change in the hydrogen release rate. Regarding the additive-promoted dehydrogenation process, the shortened incubation stage that relates to the accelerated nucleation of MgB_2_ plays an essential role, as shown in Figure 1c. In addition, the subsequent step of MgB_2_ growth that accompanies a massive amount of hydrogen release is also crucial to the improvement of the dehydrogenation kinetics of LiBH_4_–MgH_2_. This aspect is discussed in the following.

**Figure 6:**
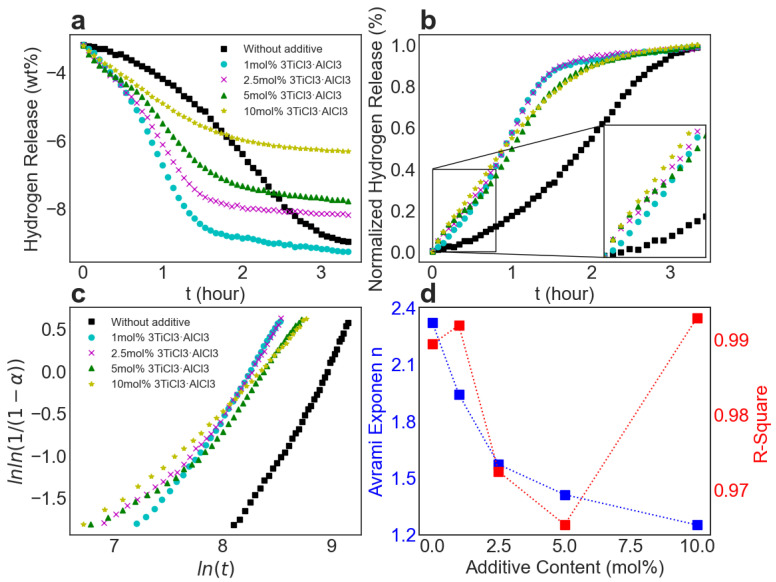
Analysis of the dehydrogenation kinetics with varying contents of additive: (**a**) Hydrogen release over time relating to the second dehydrogenation step for varying contents of additive; (**b**) Min–max normalization of the image (**a**); (**c**) JMAK plots of $\mathrm{lnln}\left(\frac{1}{1-\alpha }\right)\;\mathrm{vs}.\;\ln\left(t\right)$ with α ranging from 15% to 85% based on the image (**b**); (**d**) Change in the Avrami exponent n (left blue axis) and the corresponding R-square (right red axis) with the increase of additive content.

Figure 6a is extracted from Figure 1c and shows the hydrogen release over time for the respective samples, during the second step of the reaction, ranging from the start of the massive hydrogen release to the quasi-end of the dehydrogenation, when each curve is nearly flat. The process of counting starts with −3.2 wt% of hydrogen release to ensure the self-decomposition of MgH_2_ is completed and all the released hydrogen in this case is coming from the reaction between LiBH_4_ and Mg.

Figure 6b exhibits the min–max normalized hydrogen release between zero and one for each sample. Notably, it takes more than twice the time to release about 80% of hydrogen from the sample without additives as it does with additives. This means that both the nucleation and the growth of MgB_2_ platelets are faster than those of MgB_2_ bars. It is thus intuitive to consider a positive correlation between the hydrogen release rate and the amount of 3TiCl_3_·AlCl_3_. However, after approximately 50% of hydrogen was released, the hydrogen release rate of the sample with 10 mol% additives was smaller than that of the samples with smaller amounts of additives. One reasonable interpretation is that the mutual impingement between the generated phases becomes even more furious, as more nucleation centers of TiB_2_ exist in the surrounding environment, which inversely decelerates the dehydrogenation process. This might also be an indication of an oversaturation of the sample with additives. Nonetheless, it is still important to study extreme cases so that the physical mechanisms behind the growth of MgB_2_ can be distinguished and clarified.

The Johnson–Mehl–Avrami–Kolmogorov (JMAK) equation generally describes the kinetics of phase transformation for solids. To obtain an in-depth understanding of the dehydrogenation kinetics and determine the rate-controlling step for the MgB_2_ growth for the respective samples, this equation was applied to model the process of the MgB_2_ growth; see Equation (2) [32,33]:

(2) $$\alpha =1-\exp\left[-{\left(\mathrm{kt}\right)}^{n}\right]$$

where α denotes the fraction of released hydrogen over time (representing the volume fraction of the directly correlated MgB_2_ phase), k denotes the reaction rate constant that depends on temperature and n refers to the Avrami exponent. The numerical value of the Avrami exponent n can be regarded as an indicator of the growth dimensionality for MgB_2_ crystals and the related rate-controlling steps [32,34]. In general, n is equal to d/m, where d represents the dimensionality of crystal growth with the conditions that 1≤d ≤2 refers to one-dimensional growth (e.g., needle), 2≤d ≤3 refers to two-dimensional growth (e.g., platelet and sheet), and 3≤d ≤4 refers to three-dimensional growth (e.g., sphere). The value of m indicates the rate-controlling step for the phase transformation, with m=1 referring to the interface-controlled growth, and m=2 representing the diffusion-controlled growth. To determine n for each sample, we can rewrite Equation (2) as:

(3) ln[−ln(1−α)]=nln(t)+nln(k) 

From the equation, n can be immediately determined from the slope by plotting ln[−ln(1−α)] against ln(t). To keep the analysis consistent, the data with hydrogen release α ranging from 15% to 85% were selected for the plot of ln[−ln(1−α)] vs. ln(t); see Figure 6c. The slope is determined by linear regression with the corresponding R-square value evaluating the quality of the fit for the respective samples, as shown in Figure 6d. All the fitting parameters can be found in Table S1. As can be seen, with the increase in additive content, the Avrami exponent decreases.

Based on TEM observations of bar-like and platelet-like MgB_2_ crystals, the MgB_2_ growth was determined to be mainly two-dimensional, as the morphology extension in the third dimension along the c axis is almost negligible. The value of dimensionality d  is therefore located between two and three. From this perspective, m=1 and m=2 can be assigned to the two most extreme cases: without additives (n=2.32) and with 10 mol% additives (n=1.25), since only one MgB_2_ morphology exists for either case. Based on these values, the growth rate-controlling steps were determined to be mainly interface-controlled or mainly diffusion-controlled, respectively. The change in the growth rate-controlling step is also in agreement with the discussed decrease in the elastic strain energy density at the interface between MgB_2_ and TiB_2_ compared with that between MgB_2_ and Mg during the formation of MgB_2_. For the samples with a lower additive content, where both MgB_2_ bars and platelets were observed, it can therefore be expected that the interface-controlled and the diffusion-controlled growth may affect the dehydrogenation process simultaneously. Their Avrami exponents are thus more likely to reflect the simultaneous contribution from both growth mechanisms with varying weights for each case. This can be additionally confirmed by the decrease in their R-square values, indicating a worsened fit. When increasing the additive content up to 10 mol%, the R-square value improved again. This can be explained by the dominance of the diffusion-controlled growth, which is in agreement with the fact that only MgB_2_ platelets have been observed in this case.

Based on these results, the best additive content that accelerates the dehydrogenation process the most can be determined. It may lay between 2.5 mol% and 5 mol% additive content. According to the interpretation, this is because the growth rate-controlling step changes from interface-controlled for 2.5 mol% additives to diffusion-controlled for 5 mol% additives (Avrami exponent n<1.5). In this range, the formation of MgB_2_ platelets becomes gradually dominant. The transition in the growth rate-controlling step is also consistent with the TEM observations, where much fewer MgB_2_ bars were found when the additive content was increased to 5 mol%.

**Figure 7:**
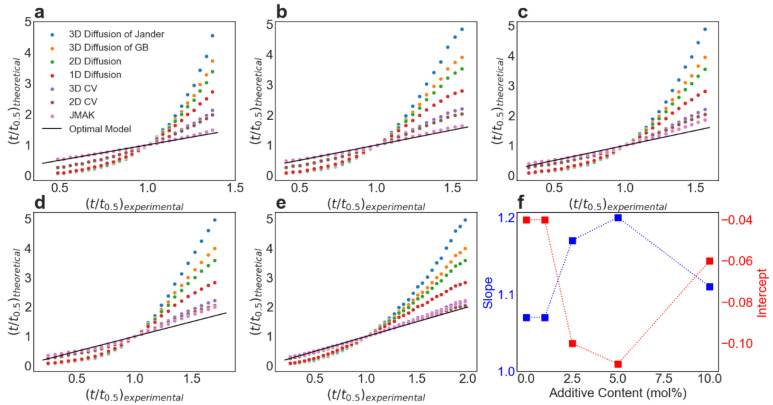
Comparison of different models for kinetic analysis: (**a**–**e**) Plots of (t/t_0.5_)_experimental_ vs. (t/t_0.5_)_theoretical_ based on the reduced time method for a variety of kinetic models in different scenarios with varying contents of additives (0, 1 mol%, 2.5 mol%, 5 mol% and 10 mol%). The optimal fitting is represented by the straight line for reference; (**f**) The corresponding slope and intercept for the JMAK model (with different Avrami exponents) according to the plots (**a**–**e**) indicates that the JMAK model describes the experimental data the best.

To further validate the results of the JMAK equation, several other frequently utilized models to study the kinetics of hydrogen storage materials were utilized for comparison; see Table 3. The fit quality of these models was evaluated by the reduced time method, by plotting the theoretical reduced time ${\left(t/t_{0.5}\right)}_{\mathrm{theoretical}}\;$ against the experimental reduced time ${\left(t/t_{0.5}\right)}_{\mathrm{experimental}}$, where $t_{0.5}$ denotes the time when 50% of hydrogen has been released [35,36]. Figure 7a–e shows the plots of ${\left(t/t_{0.5}\right)}_{\mathrm{theoretical}}\;\mathrm{vs}.\;{\left(t/t_{0.5}\right)}_{\mathrm{experimental}}$ of all these kinetic models for each sample using the data with the hydrogen release α ranging from 15% to 85%. The fitting parameters can be found in Tables S2–S6. The optimal fitting has a slope equal to one and an intercept equal to zero, so a straight line of y=x is drawn on each figure for reference. In Figure 7f, the respective intercept and slope values for the JMAK model are summarized. In general, the JMAK model performs best in fitting the data to describe the second reaction step of dehydrogenation process of LiBH_4_–MgH_2_ over time.

## 3. Materials and Methods

### 3.1. Material Preparations

The reactants were purchased in the form of powder: MgH_2_ (95% purity, Rockwood Lithium GmbH), LiBH_4_ (95% purity, Sigma-Aldrich), and 3TiCl_3_·AlCl_3_ (about 76–78% TiCl_3_ purity, Fischer Scientific). The LiBH_4_–MgH_2_ composite was mixed with a molar content of x% 3TiCl_3_·AlCl_3_ (x = 0, 1, 2.5, 5 and 10). To achieve a fine mixing of the reactants and an even dispersion of the additives, the prepared material mixtures (about 3 g), namely 2LiBH_4_–MgH_2_ or 2LiBH_4_–MgH_2_–3TiCl_3_·AlCl_3_, were charged into stainless steel vials with stainless steel balls in a ball to powder ratio of 20:1. The milling proceeded for 400 min using a Spex 8000M Mixer Mill. Both the powder handling and milling were performed under an argon atmosphere in a glovebox (O_2_, H_2_O < 0.5 ppm).

### 3.2. Kinetics Measurements

The volumetric measurements of hydrogen release were performed using a custom-built Sievert’s-type apparatus. The milled sample (~170 mg) was charged into the stainless steel sample holder of the measuring apparatus. The samples were annealed from room temperature to 400 °C at a heating rate of 10 °C min^−1^ under a hydrogen atmosphere of 4 bar. After reaching the target temperature of 400 °C, the materials were kept under isothermal conditions for several hours.

### 3.3. XRD Experiments

The ex situ XRD experiments were performed using a Bruker D8 Discover diffractometer equipped with a Cu X-ray source (λ = 1.54184 Å) and a 2D VANTEC detector. The operating voltage and current were 50 kV and 1000 mA, respectively. The diffraction patterns were acquired in the 2θ range from 10° to 90° with a step size of 0.005°, Δ2θ = 10° and the exposure time for each step of 400 s. To prevent oxidation of the materials, the specimens were packed onto a sample holder and sealed with an airtight dome made of poly (methyl methacrylate) (PMMA), which was carried out in an argon-filled glovebox (O_2_, H_2_O < 0.5 ppm).

### 3.4. TEM Experiments

TEM experiments were performed using a Themis-Z 60-300 (Thermo Fisher Scientific Inc., Waltham, MA, USA) equipped with a monochromator and double aberration correctors (probe and image Cs correctors) operated at 300 kV. TEM sample preparation was carried out under an argon atmosphere in a glovebox (O_2_, H_2_O < 0.5 ppm). Sample powders were dispersed in toluene and ultra-sonicated for 1 min before being distributed on lacey carbon-coated gold TEM grids with the item number S166-A3-V (Plano GmbH). Subsequently, they were transferred under argon from the glovebox into the microscope with a vacuum transfer holder (model number 648, AMETEK Gatan Inc.).

The utilized beam current ranged from 50 to 150 pA for TEM experiments. Selected area electron diffraction (SAED) patterns were collected using an OneView camera (AMETEK Gatan Inc.). Scanning TEM (STEM) images were recorded via a high-angle annular dark-field (HAADF) detector with a convergence angle of 21.5 mrad and a camera length of 93 mm. Energy-dispersive X-ray spectroscopy (EDX) spectrum-imaging (SI) was executed with a Super-X windowless EDX detector (Thermo Fisher Scientific Inc.) using the same parameters for STEM imaging.

Electron tomography was carried out using a Fischione 2020 tomography holder in STEM mode with a convergence angle of 8.58 mrad. Other parameters stayed the same as mentioned above. HAADF-STEM tilt series with image dimensions of 1024 × 1024 pixels were collected using Xplore3D (Thermo Fisher Scientific Inc.) over a tilt range with an increment of 2° from −70° to 70° for the desorbed 2LiBH_4_–MgH_2_ with 1 mol% 3TiCl_3_·AlCl_3_, from −74° to 76° for the desorbed 2LiBH_4_–MgH_2_ with 5 mol% 3TiCl_3_·AlCl_3_ and from −66° to 72° for the desorbed 2LiBH_4_–MgH_2_ with 10 mol% 3TiCl_3_·AlCl_3_. Electron tomography combined with EDX mapping was also carried out on the desorbed 2LiBH_4_–MgH_2_ with 10 mol% 3TiCl_3_·AlCl_3_ with a tilt range from −66° to 69° with 3° increments. Each EDX map of Mg was constructed from 900 frames with image dimensions of 256 × 256 pixels and a dwell time of 12 µs. The alignment of the tilt series was performed in IMOD using the cross-correlation method. The aligned tilt series were then reconstructed using the algorithm of simultaneous iterative reconstruction technique (SIRT) with 100 iterations in Inspect3D (Thermo Fisher Scientific Inc.) [39]. The 3D visualization was realized using Avizo 2020.2 (Thermo Fisher Scientific Inc.).

4D-STEM measurements were carried out in µ-probe mode using the OneView camera, with a convergence angle of 0.47 mrad, a camera length of 720 mm, an acquisition time of 13.2 ms for each pixel, and a dose of ~4.7 × 10^7^ e nm^−2^. STEM electron energy-loss spectroscopy (EELS) SI was acquired using a Continuum 970 HighRes imaging filter (GIF) (AMETEK Gatan Inc.) in dual-EELS mode with 13.64 ms acquisition time for each low-loss spectrum, 136.4 ms for each high-loss spectrum, 21.5 mrad convergence angle, 40 mrad collection angle, and 0.15 eV per channel energy dispersion. The EELS SI data were denoised by principal component analysis (PCA), which effectively reduces the random noise generated during signal recording [40,41].

## 4. Summary

The second dehydrogenation step of the reactive hydride composite LiBH_4_–MgH_2_ is controlled by the nucleation and growth of MgB_2_ in respective orientations. The observed different MgB_2_ morphologies can be directly correlated to the required elastic strain energy per unit volume. The nucleation of MgB_2_ on Mg requires an energy up to 2.9 × 10^8^ J/m^3^ in the $\langle 10\bar{1}0\rangle$ direction, whereas it needs only 4.7·10^7^ J/m^3^ for the nucleation of MgB_2_ on TiB_2_ in the $\langle 10\bar{1}0\rangle$ or $\langle 1\bar{2}10\rangle$ directions. The formation of MgB_2_ may occur primarily on those TiB_2_ nanoparticles that adhere to Mg grains, which leads to the generation of parallel MgB_2_ platelets. According to the JMAK equation parameter interpretation, the growth rate-controlling steps for MgB_2_ bars or platelets are interface-controlled or diffusion-controlled, respectively. The change in the growth mechanism is consistent with the decreased elastic strain energy density determined for the nucleation of MgB_2_ on TiB_2_ and the change in the morphology of MgB_2_ when additives were included. Based on the second dehydrogenation growth kinetics, the best additive content that accelerates the dehydrogenation process of LiBH_4_–MgH_2_ the most is between 2.5 mol% and 5 mol%. However, given the consumption of LiBH_4_ by additives, the trade-off between a reduced hydrogen storage capacity and improved kinetics also needs to be carefully considered in practice.

## Figures and Tables

**Table 1:** Orientation relationship between MgB_2_ and Mg or TiB_2_, and their corresponding atomic misfits [19].

	$\mathrm{Interatomic}\;\mathrm{Planes},\;\mathrm{Misfit}\;\delta_{hkil}^{2}$	$\mathrm{Interatomic}\;\mathrm{Directions},\;\mathrm{Misfit}\;\delta_{hkil}^{2}$
**MgB_2_ on Mg**	$\left\{0002\right\}\;|\;\left\{1\bar{2}10\right\}$, −9.3%	$\langle 10\bar{1}0\rangle \;||\;\langle 10\bar{1}0\rangle$, 4.2%
**MgB_2_ on TiB_2_**	{0002} | {0002}, −8.9%	$\langle 10\bar{1}0\rangle \;||\;\langle 10\bar{1}0\rangle$, −1.7%
		$\langle 1\bar{2}10\rangle \;||\;\langle 1\bar{2}10\rangle$, −1.7%

**Table 2:** The Young’s modulus and the elastic strain energy densities for MgB_2_ nucleating in the directions 0002, $10\bar{1}0$ and $1\bar{2}10$ on the respective nucleation centers Mg and TiB_2_.

Lattice Plane	0002	$10\bar{1}0$	$1\bar{2}10$
$Y_{hkl}$ **(GPa)**	184.7	326.9	326.9
$\epsilon_{hkl}$ **(J/m^3^)**	7.4 × 10^8^ (on Mg) 7.3 × 10^8^ (on TiB_2_)	2.9 × 10^8^ (on Mg) 4.7 × 10^7^ (on TiB_2_)	4.7 × 10^7^ (on TiB_2_)

**Table 3:** The introduction to the applied kinetic models [32,34,37,38].

Model	k*t=
Two-dimensional growth of contracting volume (2D CV)	$1-{\left(1-\;\alpha \right)}^{1/2}$
Three-dimensional growth of contracting volume (3D CV)	$1-{\left(1-\;\alpha \right)}^{1/3}$
One-dimensional diffusion (1D Diffusion)	$\alpha^{2}$
Two-dimensional diffusion (2D Diffusion)	[(1− α)ln(1− α)]+ α
Three-dimensional diffusion of Ginstling-Braunshtein equation (3D Diffusion of GB)	$1-\frac{2}{3}\alpha \;-{\left(1-\;\alpha \right)}^{2/3}$
Three-dimensional diffusion of Jander equation (3D Diffusion of Jander)	${\left(1-{\left(1-\;\alpha \right)}^{1/3}\right)}^{2}$

## Data Availability

The data that support the findings of this study are openly available in KITOpen at https://publikationen.bibliothek.kit.edu/1000150744. (access on 31 August 2022).

## Supplementary Materials

The following supporting information can be downloaded at: https://www.mdpi.com/article/10.3390/molecules27207005/s1.

## Sample Availability

Samples of the compounds are available from the authors.
