# Supplementary material for: Transformation Kinetics of LiBH4–MgH2 for Hydrogen Storage
Source: Molecules. 2022 Oct 18;27(20):7005. doi: 10.3390/molecules27207005 (PMC9606854; doi:10.3390/molecules27207005)
Supplement: Supplementary file 1 [file molecules-27-07005-s001.zip › molecules-1951851-supplementary.pdf]

Supplementary Information

Ou Jin [1,2], Yuanyuan Shang [3], Xiaohui Huang [2], Dorothée Vinga Szabó [1,2,4], Thi Thu Le [3], Stefan Wagner [1], Thomas Klassen [3], Christian Kübel [2,4,5], Claudio Pistidda [3] and Astrid Pundt [1,2,*]

[1] Institute for Applied Materials, Karlsruhe Institute of Technology, 76131 Karlsruhe, Germany

[2] Institute of Nanotechnology, Karlsruhe Institute of Technology, 76344 Eggenstein-Leopoldshafen, Germany

[3] Institute of Hydrogen Technology, Helmholtz-Zentrum Hereon GmbH, 21502 Geesthacht, Germany

[4] Karlsruhe Nano Micro Facility, Karlsruhe Institute of Technology, 76344 Eggenstein-Leopoldshafen, Germany

[5] Joint Research Laboratory Nanomaterials, Technical University of Darmstadt, 64206 Darmstadt, Germany

* Correspondence: astrid.pundt@kit.edu

Table S1. Summary of linear fit for Figure 6c.

		Value	Standard Error	t-Value	Adj. R^2
**Without additives**	Slope	2.3218	0.0053	438.4318	0.9894
	Intercept	-20.7894	0.0462	-449.8248	
**1 mol% $3TiCl_3{\cdot}AlCl_3$**	Slope	1.94479	0.0049	394.6992	0.9919
	Intercept	-16.0415	0.0396	-405.1646	
**2.5 mol% $3TiCl_3{\cdot}AlCl_3$**	Slope	1.5735	0.0072	219.9004	0.9724
	Intercept	-12.9968	0.0569	-228.5201	
**5 mol% $3TiCl_3{\cdot}AlCl_3$**	Slope	1.4098	0.0064	221.4647	0.9650
	Intercept	-11.8189	0.0514	-229.9530	
**10 mol% $3TiCl_3{\cdot}AlCl_3$**	Slope	1.2536	0.0024	515.1332	0.9929
	Intercept	-10.4434	0.0197	-529.7346	

Table S2. Summary of linear fit for Figure 7a.

Without additives		Value	Standard Error	t-Value	Adj. R^2
**JMAK**	Slope	1.0713	0.0026	416.9742	0.9882
	Intercept	-0.0398	0.0025	-15.8359	
**2D CV**	Slope	1.9734	0.0076	261.5305	0.9705
	Intercept	-0.8774	0.0074	118.6264	
**3D CV**	Slope	2.1321	0.0098	218.0556	0.9582
	Intercept	-1.0060	0.0096	-104.9686	
**1D Diffusion**	Slope	3.0743	0.0176	174.3302	0.9360
	Intercept	-1.8405	0.0173	-106.4796	
**2D Diffusion**	Slope	3.7143	0.0279	133.1747	0.8952
	Intercept	-2.3385	0.0273	-85.5417	
**3D Diffusion of Jander**	Slope	4.0216	0.0333	120.9172	0.8768
	Intercept	-2.5837	0.0326	-79.2806	
**3D Diffusion of GB**	Slope	4.7302	0.0464	102.0251	0.8351
	Intercept	-3.1311	0.0454	-68.9223	

Table S3. Summary of linear fit for Figure 7b.

1 mol% $3TiCl_3{\cdot}AlCl_3$		Value	Standard Error	t-Value	Adj. R^2
**JMAK**	Slope	1.0716	0.0023	469.2585	0.9942
	Intercept	-0.0420	0.0024	-17.3204	
**2D CV**	Slope	1.6666	0.00528	315.5565	0.9873
	Intercept	-0.5961	0.0056	-106.3089	
**3D CV**	Slope	1.8184	0.0072	251.3369	0.9802
	Intercept	-0.7168	0.0077	-93.3239	
**1D Diffusion**	Slope	2.6390	0.0140	188.0807	0.9651
	Intercept	-1.4365	0.0149	-96.4374	
**2D Diffusion**	Slope	3.2660	0.0236	138.6150	0.9376
	Intercept	-1.9148	0.0250	-76.5491	
**3D Diffusion of GB**	Slope	3.5784	0.0288	124.0579	0.9233
	Intercept	-2.1518	0.0306	-70.2666	
**3D Diffusion of Jander**	Slope	4.3062	0.0420	102.5285	0.8916
	Intercept	-2.7030	0.0446	-60.6187	

Table S4. Summary of linear fit for Figure 7c.

**2.5 mol% $3TiCl_3 \cdot AlCl_3$**		**Value**	**Standard Error**	**t-Value**	**Adj. R^2**
**JMAK**	Slope	1.1753	0.0049	239.0752	0.9764
	Intercept	-0.1017	0.0050	-20.2538	
**2D CV**	Slope	1.4729	0.0063	235.8112	0.9758
	Intercept	-0.3801	0.0064	-59.6064	
**3D CV**	Slope	1.5984	0.0082	196.1993	0.9654
	Intercept	-0.4742	0.0083	-57.0070	
**1D Diffusion**	Slope	2.3142	0.0148	155.9134	0.9463
	Intercept	-1.0811	0.0152	-71.3463	
**2D Diffusion**	Slope	2.8262	0.0237	119.0725	0.9113
	Intercept	-1.4436	0.0242	-59.5761	
**3D Diffusion of GB**	Slope	3.0783	0.0285	107.8588	0.8940
	Intercept	-1.6204	0.0291	-55.6114	
**3D Diffusion of Jander**	Slope	3.6618	0.0403	90.7684	0.8565
	Intercept	-2.0280	0.0412	-49.2397	

Table S5. Summary of linear fit for Figure 7d.

5 mol% $3TiCl_3{\cdot}AlCl_3$		Value	Standard Error	t-Value	Adj. R^2
**JMAK**	Slope	1.2021	0.0041	295.9564	0.9800
	Intercept	-0.1170	0.0044	-26.6525	
**2D CV**	Slope	1.3418	0.0042	321.6943	0.9830
	Intercept	-0.2609	0.0045	-57.8550	
**3D CV**	Slope	1.4632	0.0054	269.5095	0.9760
	Intercept	-0.3491	0.0059	-59.4891	
**1D Diffusion**	Slope	2.1187	0.0103	206.2832	0.9596
	Intercept	-0.8960	0.0111	-80.7042	
**2D Diffusion**	Slope	2.6196	0.0165	158.3412	0.9334
	Intercept	-1.2388	0.0179	-69.2739	
**3D Diffusion of GB**	Slope	2.8694	0.0200	143.1677	0.9197
	Intercept	-1.4085	0.0217	-65.0123	
**3D Diffusion of Jander**	Slope	3.4520	0.0288	119.8256	0.8891
	Intercept	-1.8032	0.0311	-57.9059	

Table S6. Summary of linear fit for Figure 7e.

10 mol% $3TiCl_3{\cdot}AlCl_3$		Value	Standard Error	t-Value	Adj. R^2
**JMAK**	Slope	1.1063	0.0019	583.6405	0.9945
	Intercept	-0.0656	0.0024	-27.7279	
**2D CV**	Slope	1.0762	7.5715E-4	1421.4401	0.9991
	Intercept	-0.0624	9.4477E-4	-66.0712	
**3D CV**	Slope	1.1816	0.0016	747.8207	0.9966
	Intercept	-0.1451	0.0020	-73.5863	
**1D Diffusion**	Slope	1.7341	0.0040	436.3873	0.9901
	Intercept	-0.6310	0.0050	-127.2555	
**2D Diffusion**	Slope	2.1737	0.0085	256.9680	0.9720
	Intercept	-0.9590	0.0106	-90.8541	
**3D Diffusion of GB**	Slope	2.3933	0.0111	215.8097	0.9608
	Intercept	-1.1223	0.0138	-81.1047	
**3D Diffusion of Jander**	Slope	2.9053	0.0178	163.5313	0.9337
	Intercept	-1.5035	0.0222	-67.8186	
